# Nanoflower-like P-doped Nickel Oxide as a Catalytic Counter Electrode for Dye-Sensitized Solar Cells

**DOI:** 10.3390/nano12224036

**Published:** 2022-11-17

**Authors:** Yi-Lin Chen, Yi-June Huang, Min-Hsin Yeh, Miao-Syuan Fan, Cheng-Tai Lin, Ching-Cheng Chang, Vittal Ramamurthy, Kuo-Chuan Ho

**Affiliations:** 1 Department of Chemical Engineering, National Taiwan University, Taipei 10617, Taiwan; 2 Department of Chemical Engineering, National Taiwan University of Science and Technology, Taipei 10607, Taiwan; 3 Institute of Polymer Science and Engineering, National Taiwan University, Taipei 10617, Taiwan

**Keywords:** counter electrode, dye-sensitized solar cell, flower-like morphology, phosphorus-doped nickel oxide, rear illumination

## Abstract

Flower-like phosphorus-doped nickel oxide (P-NiO) is proposed as a counter electrode (CE) for dye-sensitized solar cells (DSSCs). The flower-like nickel oxide essentially serves as the matrix for the CE, which is expected to promote a two-dimensional electron transport pathway. The phosphorus is intended to improve the catalytic ability by creating more active sites in the NiO for the catalysis of triiodide ions (I_3_^−^) to iodide ions (I^−^) on the surface of the CE. The P-NiO is controlled by a sequencing of precursor concentration, which allows the P-NiO to possess different features. The debris aggregation occurs in the P-NiO-1, while the P-NiO-0.75 leads to the incomplete flower-like nanosheets. The complete flower-like morphology can be observed in the P-NiO-0.5, P-NiO-0.25 and P-NiO-0.1 catalytic electrodes. The DSSC with the P-NiO-0.5 CE achieves a power conversion efficiency (η) of 9.05%, which is better than that of the DSSC using a Pt CE (η = 8.51%); it also performs better than that with the Pt CE, even under rear illumination and dim light conditions. The results indicate the promising potential of the P-NiO CE to replace the expensive Pt CE.

## 1. Introduction

With the growing threat of an energy crisis, renewable energy is becoming more important around the world. Dye-sensitized solar cells (DSSCs) offer commercial possibility and attract research attention in third-generation photovoltaic devices due to their low cost, simple fabrication, broad spectral absorption range and environmental friendliness [1,2,3,4]. Progress has been made on DSSCs since O’Regan and Grätzel reported a breakthrough power conversion efficiency (η) of 7.12% [5]. Nowadays, the best performance of DSSCs reaches 13.0% cell efficiency, which is displayed in NREL’s Best Research-Cell Efficiencies Chart [6]. The DSSC is composed of the photoanode (dye and oxides), the electrolyte and the counter electrode (CE). In addition to being a necessary component of a DSSC, the CE plays a significant role in determining how well it will function. It should have a low charge transfer resistance and excellent catalytic activity for the conversion of triiodide ions (I_3_^−^) to iodide ions (I^−^) at the CE/electrolyte interface. Facile I_3_^−^ reduction kinetics at the CE leads to minimum charge transfer resistance. Achieving a high solar-to-electricity conversion efficiency for the DSSC while also lowering its cost becomes a key issue in the development of DSSCs. Platinum (Pt) has been known to exhibit outstanding electro-catalytic activity toward the I_3_^−^/I^−^ redox couple as well as high electrical conductivity while acting as the CE [7]. However, Pt is scarce and expensive, which makes it difficult for cost-effective fabrication in DSSCs, especially in large-scale productions [8]. Carbon materials [9,10,11,12,13], conducting polymers [14,15] and inorganic transition metal compounds [16,17,18] have been investigated as the substitutes for Pt to reduce the cost of production in DSSCs.

Among them, transition metal compounds, such as sulphides [19,20], nitrides [21,22], oxides [23,24], phosphides [25,26] and selenides [27,28] have shown excellent electro-catalytic ability for the reduction of I_3_^−^ in DSSCs; they also possess good thermal stability. Nickel oxide (NiO) has become a potential material as the CE due to its high chemical stability [29] and a valence band energy (~−4.96 eV) comparable to the redox potential of I^−^/I_3_^−^ for favorable charge transfer between the metal oxide and the redox couples [30,31]. However, low conductivity of NiO prevents the extensive usage in electro-catalytic materials. Guai et al. prepared a CE film consisting of sulfur-doped NiO with a nanorod structure; the pertinent DSSC showed an η of 5.04%, which is close to that of a cell with a conventional Pt electrode [30]. Phosphorus (P)-doped reduced graphene oxide (rGO) was employed as the CE material in a DSSC by Wang et al.; they observed that the doping of P effectively enhanced the electro-catalytic activity of the rGO and found that the DSSC with this CE exhibited a comparable electro-catalytic activity to that of a Pt CE [32]. Dual-doping of nitrogen (N) and P heteroatoms in graphene was found to markedly improve the photovoltaic performance of DSSCs by a synergistic effect; a high conversion efficiency of 8.57% was achieved by Yu et al. [33].

Here, we synthesized the flower-like morphology of P-NiO by the solvothermal method and used it as the catalytic film on the CE of a DSSC. The P doping was expected to increase the active sites in the film [32,33], and the NiO was envisaged to provide a higher reaction area for the P-NiO film, which could benefit the electro-catalytic reduction of I_3_^−^ to I^−^. The fact that P-NiO CE offers better cell performance than that of Pt CE suggests that the P-NiO exhibits great potential as a low-cost and highly efficient CE material for DSSCs. The effect of the precursor concentration on the morphology of P-NiO films and η was also investigated. Furthermore, P-NiO films were applied in dim light conditions for extensive usage.

## 2. Materials and Methods

### 2.1. Materials

Nickel (II) nitrate hexahydrate (Ni(NO_3_)_2_ • 6H_2_O), trioctylphosphine (TOP, 97%), titanium (IV) tetraisopropoxide (TTIP, >98%), 2-methoxyethanol (CH_3_OCH_2_CH_2_OH, ≥99.5%), lithium perchlorate (LiClO_4_, ≥98.0%), ethanol (EtOH, 99.5%) and isopropyl alcohol (IPA, 99.5%) were purchased from Sigma-Aldrich (St. Louis, MO, USA). Iodine (I_2_, synthetic grade), lithium iodide (LiI, synthetic grade), and poly(ethylene glycol) (PEG, MW~20,000) were supplied by Merck (Rahway, NJ, USA). Acetone (≥99%), 4-tert-butylpyridine (tBP, 96%), and tert-butyl alcohol (tBA, 96%) were acquired from Acros, and 3-methoxypropionitrile (MPN, 99%) was bought from Fluka (Buchs, Switzerland). Transparent titanium dioxide (TiO_2_) paste (TL paste, Ti-nanoxide HT/SP, 13 nm), cis-diisothiocyanato-bis(2,2′-bipyridyl-4,4′-dicarboxylato) ruthenium (II) bis(tetrabutylammonium) (N719 dye), and 1,2-dimethyl-3-propylimidazolium iodide (DMPII) were obtained from Solaronix (S.A., Aubonne, Switzerland). Commercial light-scattering TiO_2_ particles (ST-41 with 200 nm) were procured from Ishihara Sangyo, Ltd. (Nishi-ku, Osaka, Japan). Nitric acid (HNO_3_, 65% solution in water), acetonitrile (ACN, 99.99%) was received from J. T. Baker. Polyester tape (1350F-1, 63 μm) was from the 3M Company (St. Paul, MN, USA).

### 2.2. Synthesis of P-NiO

Ni(NO_3_)_2_ • 6H_2_O and TOP with a molar ratio of 2:1 were mixed together in EtOH solvent and transferred to a Teflon^®^ vessel for a solvothermal synthesis of P-NiO powder. The mixture was heated at 180 °C for 2 h in an oven and then cooled down at room temperature. The obtained solution was centrifuged at 4000 rpm for 15 min for 5 times to remove excess precursors. Finally, the product was obtained after being vacuumed overnight to eliminate the remaining solvent. The products were denoted P-NiO-1, P-NiO-0.75, P-NiO-0.5, P-NiO-0.25 and P-NiO-0.1, which correspond to the TOP concentrations 1 × 10^−3^, 0.75 × 10^−3^, 0.5 × 10^−3^, 0.25 × 10^−3^ and 0.1 × 10^−3^ M, respectively.

### 2.3. Preparation of Various CEs

Fluorine-doped tin dioxide (FTO, TEC-7, 7 Ω sq.^−1^, NSG America, Inc., (Luckey, OH, USA) was washed by several solvents, including acetone, neutral cleaner, deionized water (DI water) and isopropyl alcohol, sequentially. A Pt CE was obtained on an FTO substrate by a direct-current sputter method. The films of P-NiO were deposited on the FTO glass (1.0 cm × 1.0 cm) by drop-coating 25 μL of the slurry of P-NiO. Each slurry was obtained by dissolving 10 mg of P-NiO in 1 mL of absolute EtOH. The FTO was then heated at 60 °C to evaporate the EtOH. After that, the P-NiO CEs were annealed at 550 °C in nitrogen for 1 h. An FTO glass with a pure NiO film was also annealed at 550 °C in air.

### 2.4. Preparation of Photoanode and Fabrication of the DSSC

First, a TiO_2_ colloid was prepared by mixing 0.5 M TTIP and 0.1 M nitric acid with continuous stirring and heating simultaneously at 88 °C for 8 h. The obtained solution was transferred into an autoclave (PARR 4540, Moline, IL, USA) and the temperature was maintained at 240 °C for 12 h. A colloid of TiO_2_ was attained with uniform nanoparticle size of ca. 20 nm. The scattering layer paste (SL paste) of 8 wt% crystalline TiO_2_ nanoparticles was acquired by the addition of 25 wt% of PEG and 100 wt% of ST-41 (with respect to the weight of TiO_2_) into the previous TiO_2_ colloid. The addition of PEG was intended to avoid the aggregation of TiO_2_ nanoparticles in its film and to prevent cracking during its annealing. Second, a compact layer was coated on the FTO by spinning a mixture of TTIP and 2-methoxyethanol (weight ratio of 1:3). Lastly, a transparent layer of TiO_2_ (ca. 5 μm) was coated on the compact layer by a doctor blade technique using a commercial TL paste, and a scattering layer of TiO_2_ about 5 μm thickness was coated on the transparent layer by the same technique using the SL paste obtained above. Each TiO_2_ layer was individually sintered at 500 °C for 30 min in air. Afterwards, the TiO_2_ film was immersed in a 5 × 10^−4^ M N719 dye solution in ACN/tBA (volumetric ratio of 1:1) at room temperature for 24 h. The photoanode was prepared eventually. The DSSC was fabricated with the photocathode and the CE, separated by a polyester tape spacer of 63 μm. The electrolyte, containing 0.05 M I_2_, 0.1 M LiI, 0.6 M DMPII, and 0.5 M tBP in MPN/ACN (volumetric ratio of 1:1), was injected into the gap between the two electrodes by capillarity.

### 2.5. Characterization of the CEs and the DSSCs

X-ray diffraction (XRD) analysis of the CEs was performed by an automatic X-ray diffractometer (Rigaku, Japan). Surface morphology and element details of the films were observed by using a field emission scanning electron microscopy (FE-SEM, Nova NanoSEM 230, FEI, Oregon, USA) equipped with a component for energy dispersive analysis of X-ray (EDAX, Horiba, 7021-H). Surface chemical analyses of the films were made by X-ray photoelectron spectroscopy (XPS, Thermo Scientific Theta Probe, UK). Their X-Ray source is monochromated and microfocused Al Kα, and the peak calibration procedure is based on C1s 284.6 eV to progress. The sample was pretreated by placing in a chamber and vacuumed at 8 × 10^−9^ mbar for 12 h. Transmittance spectra of films were taken by an ultraviolet-visible near-infrared spectrophotometer (UV-Vis-NIR, JASCO V-670, Japan). Photovoltaic measurements were performed under AM1.5G with an incident light intensity of 100 mW cm^−2^, by using a class A quality solar simulator (XES-301S, AM1.5G, San-Ei Electric Co., Ltd., Osaka, Japan). To obtain the photocurrent density-voltage curves (J-V curves) of the DSSCs, a potentostat/galvanostat (PGSTAT 30, Autolab, Eco-Chemie, Utrecht, the Netherlands) was set at 100 mV s^−1^ scan rate in linear sweep voltammetry (LSV) mode. The incident light intensity was calibrated with a standard Si cell (PECSI01, Peccell Technologies, Inc., Kanagawa, Japan). 

Electro-catalytic abilities of the films for redox couple of I^−^/I_3_^−^ were quantified in a three-electrode electrochemical system through cyclic voltammetry (CV). The CV curves were obtained in the electrochemical system, containing an ACN-based solution of 10 mM LiI, 1.0 mM I_2_ and 0.1 M LiClO_4_, using a potentiostat/galvanostat (PGSTAT 30, Autolab, Eco-Chemie, Utrecht, the Netherlands). A Pt foil and an Ag/Ag^+^ electrode were used as the counter and reference electrodes, respectively. FTO glasses with the films of P-NiO-0.5, bare NiO and Pt were used as the working electrodes in CV analyses. The scan rate was set at 100 mV s^−1^ for the CV analyses. Tafel polarization curves and electrochemical impedance spectra (EIS) were acquired by using symmetrical cells. Each symmetrical cell was assembled with the same type of electrode as the anode and the cathode. To get Tafel plots and EIS spectra, the same potentiostat/galvanostat (PGSTAT 30, Autolab, Eco-Chemie, Utrecht, the Netherlands), equipped with an FRA2 module, was used. An electrolyte was made up of 0.1 M LiI, 0.6 M DMPII, 0.05 M I_2_, and 0.5 M tBP in MPN/ACN (volume ratio of 1:1) was injected into each of the symmetric cells. Incident photon-to-current conversion efficiency (IPCE) plots of the DSSCs were shown in the wavelength range of 400 to 800 nm, by using another class A quality solar simulator (PEC-L11, AM1.5G, Peccell Technologies, Inc., Kanagawa, Japan), equipped with a monochromator (model 74100, Oriel Instrument, CA, USA). The incident radiation flux (ϕ) was obtained with an optical detector (model 71580, Oriel Instrument, CA, USA) and a power meter (model 70310, Oriel Instrument, CA, USA).

## 3. Results and Discussion

### 3.1. X-ray Diffraction Patterns, Energy Dispersive Analysis of X-ray, and X-ray Photoelectron Spectra

**Figure 1:**
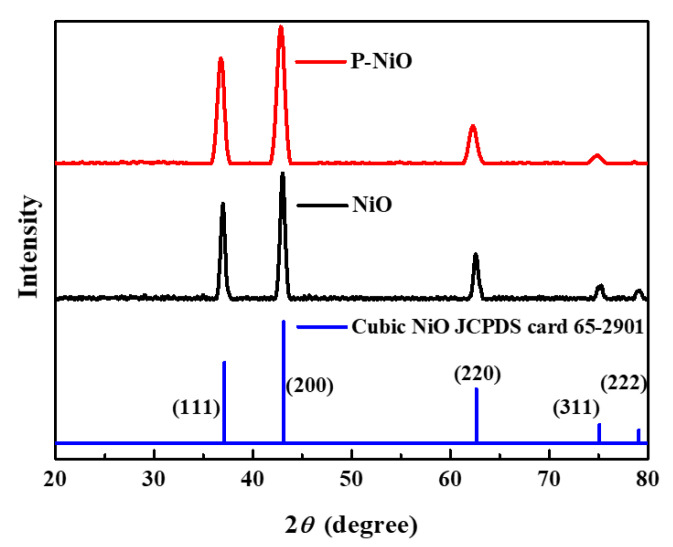
XRD patterns of the P-NiO and NiO.

**Figure 2:**
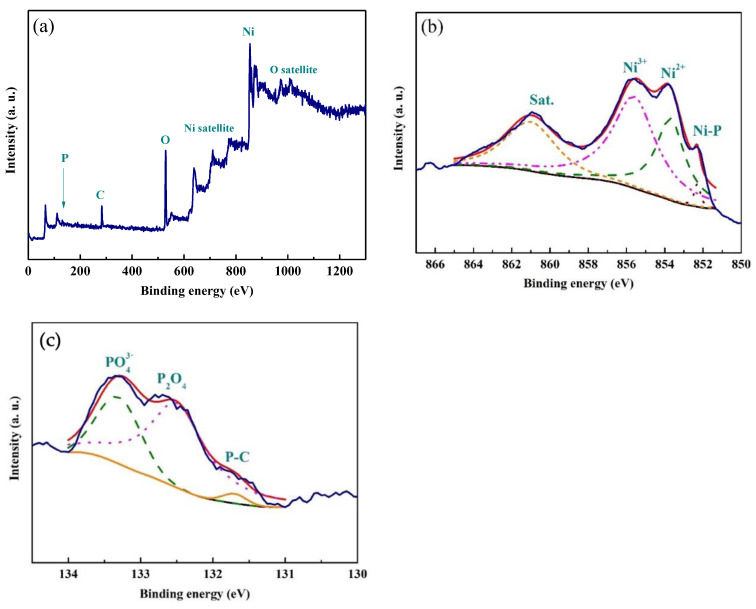
XPS spectra of the P-NiO in (**a**) the whole region, (**b**) the Ni 2p 3/2 region and (**c**) the P 2p region.

X-ray diffraction (XRD) patterns of nickel oxide (NiO) and a phosphorus-doped nickel oxide (P-NiO) are shown in Figure 1. Following the JCPDS card (No. 65-2901), the XRD patterns of NiO and P-NiO fit the cubic NiO crystal phase, which exhibit several diffraction peaks at 37.23⁰, 43.09⁰, 62.83⁰, 75.35⁰, and 79.20⁰, corresponding to the crystal planes of (111), (200), (220), (311), and (222), respectively. According to Bragg’s law, the 2 theta value would be smaller in the XRD pattern since the d-space becomes larger during the P element intercalating into the NiO crystal phase. Here, the P-NiO has almost the same diffraction peak positions as that of NiO. This reveals that few P insertions happen in the P-NiO crystal phase. In addition, we apply energy dispersive analysis of X-ray (EDAX) to identify the elements of the electrode with P-NiO, as shown in Figure S1. In the P-NiO CE, the C, O, P, Ni and Sn elements have an atomic percentage (at.%) of 10.06%, 48.39%, 0.41%, 38.19% and 2.94%, respectively. The Ni, O and P peaks are obviously recognized in the EDAX pattern of the P-NiO CE. The C peak implies that the TOP (precursor) remains in P-NiO thin film as carbon derivative after annealing. The Sn peak comes from the substrate of FTO glass. The Ni:P atomic ratio (93.15) shows that the P element is rare in the P-NiO CE, which agrees well with the results of XRD patterns. An X-ray photoelectron spectroscopy (XPS) survey spectrum is used to investigate the bonding characteristic of P-NiO CE, as shown in Figure 2a. The spectrum of Ni 2p3/2 orbital displays four peaks in Figure 2b: (1) one peak at 852.30 eV, referring to the Ni-P bonds [34,35]; (2) two peaks at 853.70 and 855.60 eV, corresponding to Ni^2+^ and Ni^3+^; (3) one peak at 861.00 eV, relating to the Ni satellite [36]. Figure S2 shows three peaks in the O 1s spectrum: (1) two peaks at 529.30 and 531.10 eV, referring to the O-Ni bonds; (2) one peak at 532.70 eV, corresponding to O-P bonds. The spectrum of P 2p orbital presents three peaks in Figure 2c: (1) one peak at 131.80 eV, corresponding to P-C bonds (2) two peaks at 132.35, 133.30 eV, referring to the P-O bonds. The Ni-P and O-P bonds indicate that the P-NiO is successfully synthesized in this study. The P-C bond implies that TOP (precursor) still exists after 550 °C in nitrogen for 1 h. In this section, the results of XRD, EDAX and XPS demonstrated that few P insertions were doped on the surface of NiO successfully.

### 3.2. Field Scanning Electron Micrographs of the Films

**Figure 3:**
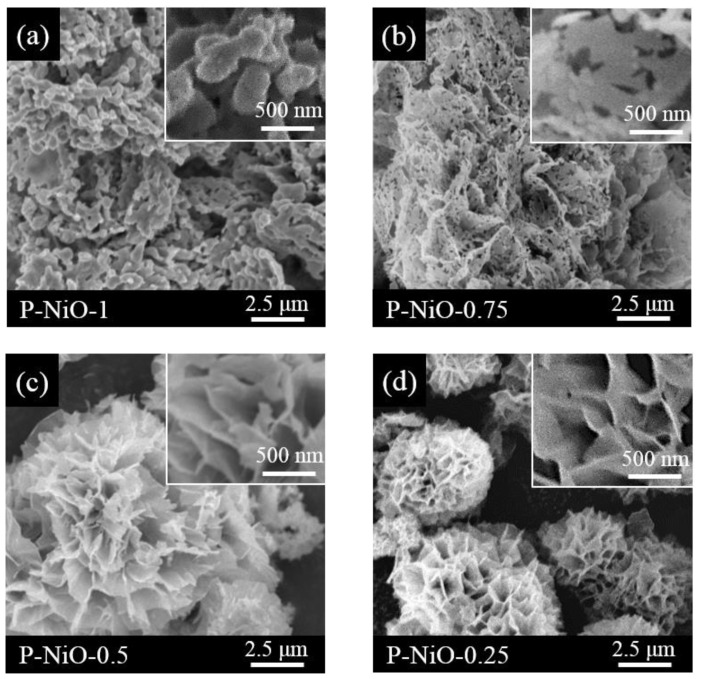

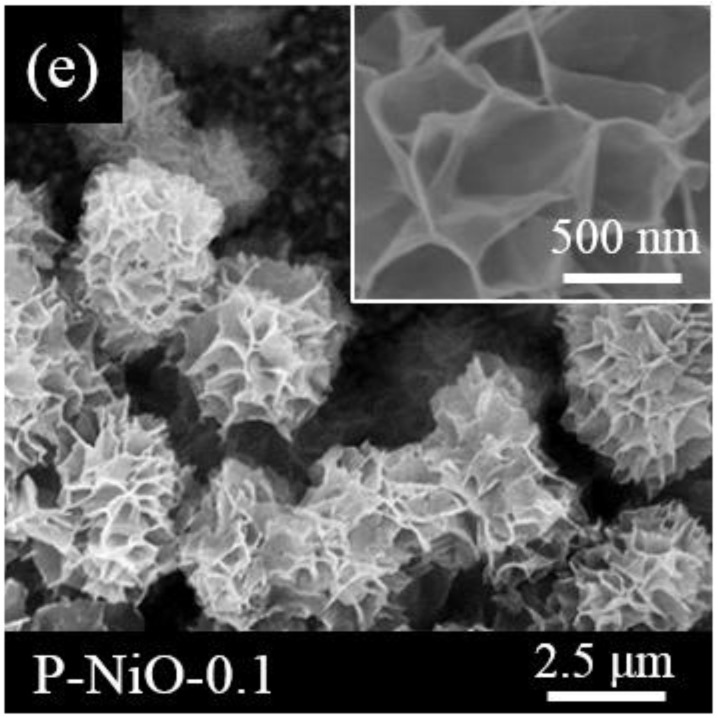
FE-SEM images of (**a**) P-NiO-1, (**b**) P-NiO-0.75, (**c**) P-NiO-0.5, (**d**) P-NiO-0.25 and (**e**) P-NiO-0.1 after the annealing process.

In this work, the P-NiO was synthesized with constant molar ratio of Ni(NO_3_)_2_ • 6H_2_O to TOP (2:1) but with different concentrations of precursors. It has been mentioned that P-NiO-1 is the product synthesized from 2 × 10^−3^ M of Ni(NO_3_)_2_ • 6H_2_O and 1 × 10^−3^ M of TOP; similarly, P-NiO-0.75, P-NiO-0.5, P-NiO-0.25 and P-NiO-0.1 correspond to TOP concentrations at 0.75 × 10^−3^, 0.5 × 10^−3^, 0.25 × 10^−3^ and 0.1 × 10^−3^ M, respectively. Figure 3 shows the field emission scanning electron microscopy (FE-SEM) of these films. Irregular particles for P-NiO-1 are shown in Figure 3a. In Figure 3b, pores are found on the flower-like nanosheets of P-NiO-0.75. From Figure 3c–e, the three films exhibit morphology of complete nanosheets, which possess thickness about 30~50 nm, and the diameters of P-NiO-0.5, P-NiO-0.25 and P-NiO-0.1 shrink sequentially. The diameters of flower-like morphology were found to be 12, 5~7 and 2.5~3 μm, corresponding to P-NiO-0.5, P-NiO-0.25 and P-NiO-0.1, respectively. The reason why there are irregular particles and incomplete flower-like nanosheets is that P-NiO grew too fast to form the P-NiO architecture, so the P-NiO architecture was shattered to a particle after the annealing process. This can be seen by comparing the FE-SEM images in Figure 3 and Figure S3. Hence, P-NiO-1 is seen as aggregate fragments and P-NiO-0.75 is shown as porous nanosheets. Moreover, the cross sections of P-NiO-1, P-NiO-0.75, P-NiO-0.5, P-NiO-0.25 and P-NiO-0.1 are shown in Figure S4. The thicknesses of P-NiO-1, P-NiO-0.75, P-NiO-0.5, P-NiO-0.25 and P-NiO-0.1 CE are about 13.4, 11.1, 9.8, 7.7 and 5.8 μm, respectively. The thickness of P-NiO increases when the precursor of TOP concentration is increased. This parameter is one of the key factors to affect the electrocatalytic ability of P-NiO CE.

### 3.3. Photovoltaic Performance of the DSSCs

**Figure 4:**
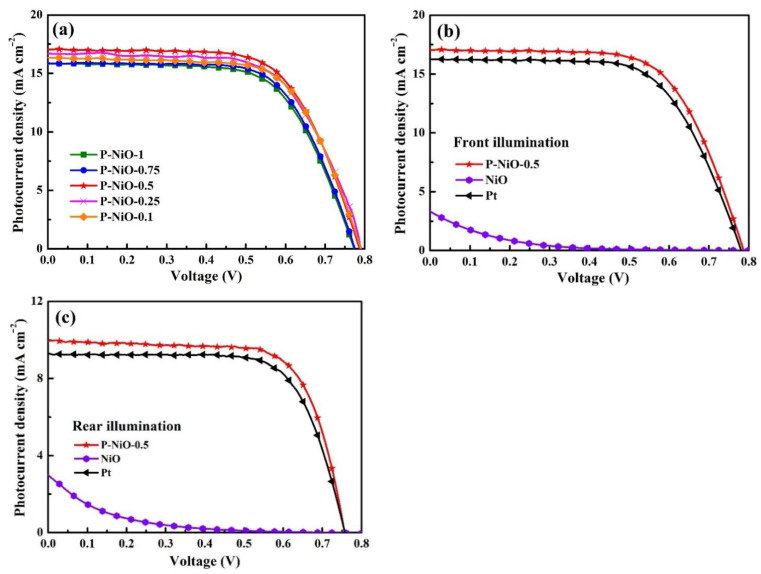
(**a**) DSSC photocurrent density-voltage curves having various P-NiO CE films illuminated in front at 100 mW cm^−2^ (AM1.5G), (**b**) photocurrent density-voltage curves of the DSSCs with P-NiO-0.5, NiO and Pt CEs illuminated in front at 100 mW cm^−2^ (AM1.5G) and (**c**) photocurrent density-voltage curves of the DSSCs with P-NiO-0.5, NiO and Pt CEs illuminated in rear at 100 mW cm^−2^ (AM1.5G).

The photocurrent density-voltage curves (J-V curves) of the DSSCs were measured at an illumination of 100 mW cm^−2^ (AM1.5G). Figure 4a illustrates the J-V curves of the DSSCs with various P-NiO films. The photovoltaic parameters, including the open-circuit voltage (*V_oc_*), short-circuit photocurrent density (*J_sc_*), fill factor (FF) and power conversion efficiency (PCE, η), of the DSSCs are listed in Table 1.

**Scheme 1:**
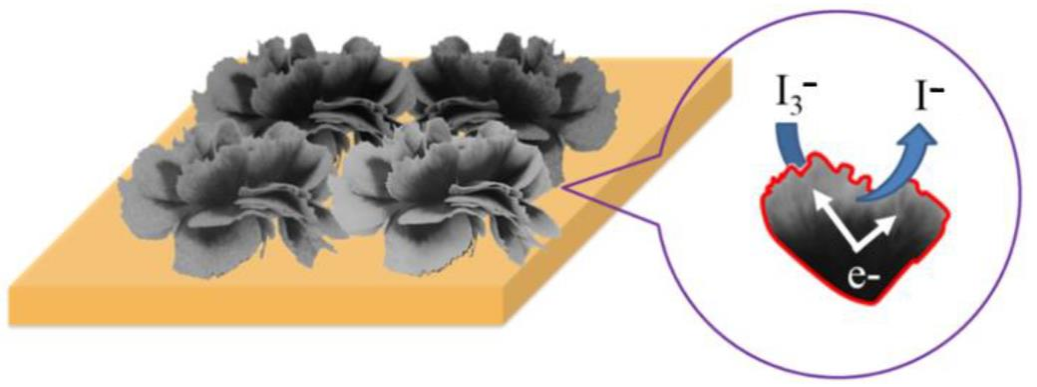
The proposed electron transfer mechanism for flower-like morphology of P-NiO.

The DSSC with P-NiO-0.5 CE achieves the best η of 9.05%, while those with P-NiO-1, P-NiO-0.75, P-NiO-0.25 and P-NiO-0.1 CE have an η of 7.95, 8.10, 8.67 and 8.54%, respectively. The tendency of *J_sc_* is the same as that of η, indicating that the η of the DSSCs depends on the values of *J_sc_*. The higher the *J_sc_*, the higher the catalytic activity for the catalysis of triiodide ions (I_3_^−^) to iodide ions (I^−^). The behavior of the *J_sc_* and the η of the DSSCs can thereby be correlated with the morphology of the CEs, as shown in FE-SEM images (Figure 3 and Figure S3). Scheme 1 illustrates that the flower-like structure provided two-dimensional space and promotes electron transfer. Thus, the reduction of I_3_^−^ occurs more easily on the flower-like structure. The aggregated structures of P-NiO-1 are unfavorable for electron transfer [37]; additionally, the porous nanosheets of P-NiO-0.75 are also the obstacle to the electron transport pathway. Since the associated surface areas appear to be smaller, the *J_sc_* in the cases of P-NiO-0.25 and P-NiO-0.1 could be smaller, thereby rendering the η of their corresponding DSSCs smaller. Obviously, the DSSC fabricated with P-NiO-0.5 film is an optimum device; we therefore choose this DSSC for our further investigation.

Figure 4b shows the J-V curves of the DSSCs with the CE films of P-NiO-0.5, bare NiO, and platinum (Pt); Table 2 gives their photovoltaic parameters. The DSSC with P-NiO-0.5 CE shows the best η of 9.05%, while the Pt CE-equipped cell produces an η value of 8.51%; this proves that the P-NiO-0.5 catalytic film is superior and can serve as an alternative CE. The DSSC with bare NiO CE only obtains an η of 0.19%, owing to the poor conductivity of NiO, as expected [38,39]; it will be proved later that NiO film has poor electro-catalytic ability. Hence, it is clear that the doped P increased the catalytic ability of NiO in a multi-fold way. We further investigated the photovoltaic performance of the above three DSSCs subjected to rear illumination. It has been noticed that the P-NiO CE is highly transparent, and the optical transmission behaviors of these films are revealed in Figure S5. Rear illumination implies that the incident light would penetrate from the side of the CE. As shown in Figure 4c and Table S1, the DSSC with P-NiO-0.5 shows the highest η of 5.45% among the three cells. The DSSC with the Pt CE shows an η of only 4.99% for rear illumination. The result indicates that the DSSC of P-NiO-0.5 CE has better performance, not only in front illumination but also in rear illumination.

### 3.4. Cyclic Voltammetry of the CEs

A key method for determining the electro-catalytic efficiency of CEs in a three-electrode setup for the reduction of I_3_^−^/I^−^ is cyclic voltammetry (CV). The CV patterns show one common pair of oxidation-reduction peaks. Equations (1) and (2), respectively, can be used to represent the anodic and cathodic peaks:

3I^−^ → I_3_^−^ + 2e^−^ (1)

I_3_^−^ + 2e^−^ → 3I^−^ (2)

**Figure 5:**
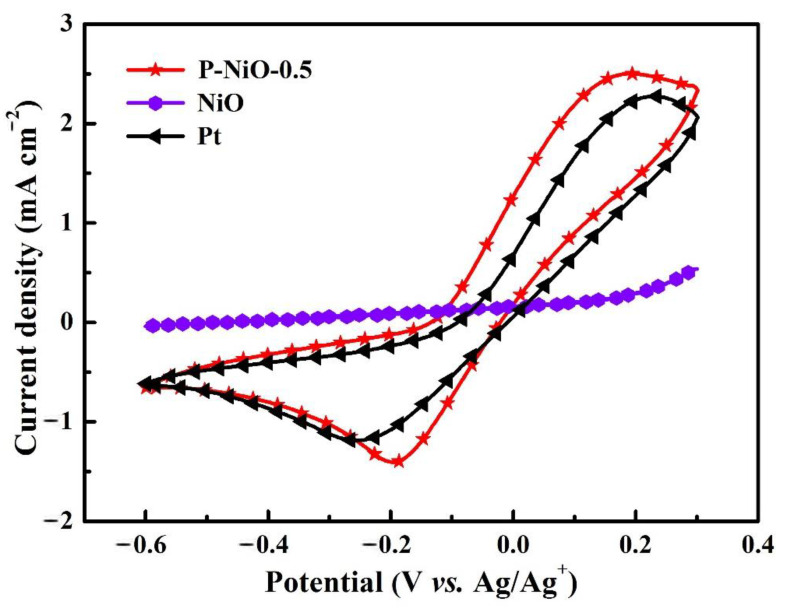
Cyclic voltammograms of P-NiO-0.5, NiO and Pt CEs at the scan rate of 100 mV/s.

Figure 5 shows the CV curves for the CEs with P-NiO-0.5, bare NiO and Pt. The values of the cathodic peak current density *(J_pc_*) and the peak separation (ΔE_p_) for these CEs are summarized in Table 3.

As they represent the electro-catalytic abilities of the CEs for the reduction of I_3_^−^, a higher *J_pc_* and a lower ΔE_p_ indicate the better electro-catalytic ability of the CE for reduction of I_3_^−^ [40,41]. Figure 5 shows the pertinent CV curve for the CE with NiO virtually represents a flat curve, so that values of *J_pc_* and ΔE_p_ could not be estimated; this happens when an electrode’s film is passive or non-faradic. The ΔE_p_ of P-NiO CE (0.38 V) is smaller than that of the Pt CE (0.47 V), suggesting that the charge transfer process is faster in the former case as compared to that in the latter case. Moreover, the *J_pc_* of P-NiO-0.5 CE is higher than that of the Pt CE. Comparing Table 2 and Table 3, it can be seen that the tendencies of *J_pc_* and ΔE_p_ are consistent with the tendency of *J_sc_* for the DSSCs with the P-NiO-0.5 and Pt CEs. To sum up, the results from the CV analysis confirm that the film of P-NiO is a better alternative to Pt for a DSSC.

### 3.5. Tafel Polarization Plots of the CEs

**Figure 6:**
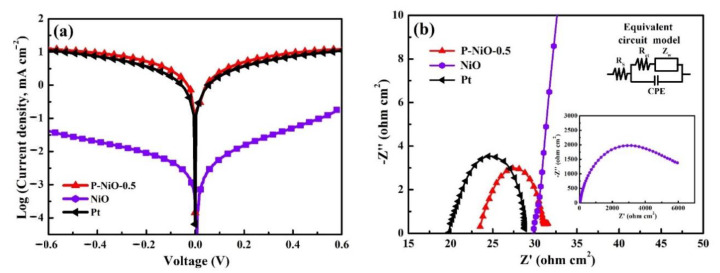
(**a**) Tafel polarization plots and (**b**) electrochemical impedance spectra of P-NiO-0.5, NiO and Pt CEs.

Tafel polarization curves are obtained to describe the electro-catalytic abilities of the CE in the reduction of I_3_^−^ by a symmetric cell. Tafel polarization plots are shown as a curve of logarithmic current density versus voltage in Figure 6a. From the Tafel plot, the exchange current density (*J_0_*) of an electro-catalytic film can be obtained by extrapolating the anodic and cathodic curves in the Tafel zone and reading the cross point at 0 V. The *J_0_* value is related to the catalytic ability of the electrode. The higher *J_0_* value represents the higher the electro-catalytic ability for a CE film. As summarized in Table 3, the *J_0_* values of the symmetric cells with P-NiO-0.5, NiO and Pt are 3.37, 4.27 × 10^−3^ and 2.71 mA cm^−2^, respectively. Since P-NiO-0.5 CE delivers the greatest *J_0_* value in its Tafel zone and the largest slope in its polarization zone, the P-NiO-0.5 CE possesses the best electro-catalytic ability among these films, even better than that of the traditional Pt CE. The *J_0_* of the film of NiO is worse, as expected from the performance of its DSSC. The *J_0_* value of a CE can be used to calculate the charge transfer resistance (R_ct-Tafel_) at the interface of the CE/electrolyte, as reflected in Equation (3):

(3) $$J_{0}=\frac{\mathrm{RT}}{{nFR}_{\mathrm{ct}\text{-}\mathrm{Tafel}}}$$

where n is the number of electrons transported during I_3_^−^ reduction, T is the absolute temperature, R is the ideal gas constant, and F is the Faraday constant. The higher the *J*_0_ value, the better electro-catalytic activity of the film; similarly, the smaller the R_ct-Tafel_ value, the better the charge transfer at the CE/electrolyte interface [42]. In Table 3, the charge transfer resistance (R_ct-Tafel_) with P-NiO-0.5 CE is smaller than that of the Pt CE; in other words, the P-NiO-0.5 film has greater electro-catalytic activity than that of the Pt film. In the case of NiO, the value of R_ct-Tafel_ cannot be compared, indicating that the film possesses huge resistance for a charge cross it.

### 3.6. Electrochemical Impendence Spectroscopy of the CEs

Electrochemical impendence spectroscopy (EIS) is utilized to investigate the interfacial resistances, series resistance (R_s_) and charge transfer resistance (R_ct-EIS_) from the higher to lower frequency in a symmetric cell, which is the same configuration as used to obtain the Tafel polarization. The R_s_ represents the contact resistance of the FTO substrate, and the R_ct-EIS_ expresses the charge transfer resistance at the CE/electrolyte interface. In Figure 6b and Table 3, the R_s_ value of the P-NiO-0.5 CE is a little higher than that of the Pt CE, indicating that the Pt film is more firmly bonded to the FTO substrate. Though the R_s_ value of the P-NiO-0.5 CE is higher than that of the Pt CE, the R_ct-EIS_ value of the former is smaller than that of the latter; therefore, the overall catalytic ability of the P-NiO-0.5 CE is still better than that of the Pt CE. The R_s_ with the NiO CE is much higher than that with the P-NiO-0.5 and Pt CEs. The NiO CE also exhibits a considerably high R_ct-EIS_ value in reference to that of the P-NiO-0.5 CE; this means that the charge transfer resistance of the P-NiO film is enormously reduced owing to the doping of P. It is notable that the values of the R_ct-EIS_ are very close to the values of the R_ct-Tafel_; furthermore, all these R_ct_ values show a perfect consistency with the values of *J_sc_* and *J_pc_*.

### 3.7. Incident Photon-to-Current Conversion Efficiency (IPCE) Spectra of the DSSCs

**Figure 7:**
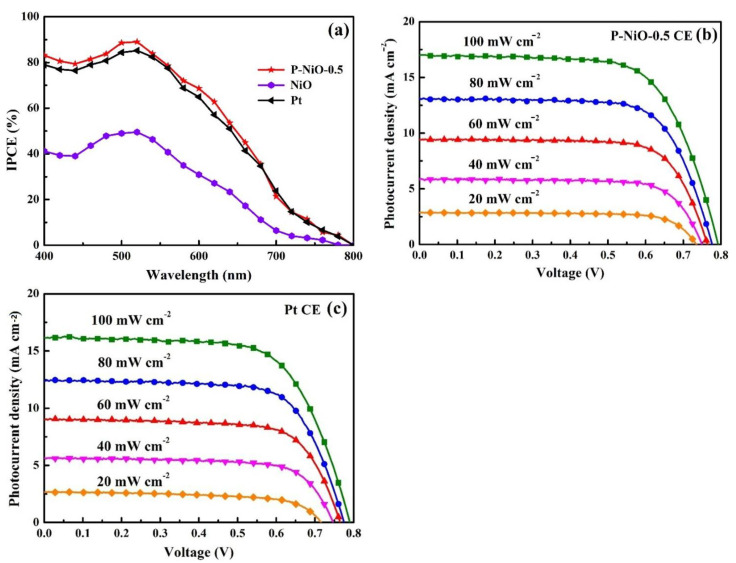
(**a**) IPCE curves of the DSSCs with P-NiO-0.5, NiO and Pt CEs, (**b**) photocurrent density-voltage curves of the DSSCs with the P-NiO-0.5 CE obtained at different light conditions and (**c**) photocurrent density-voltage curves of the DSSCs with the Pt CE obtained at different light conditions.

Incident photon-to-current conversion efficiency plots (IPCE) spectra of the DSSCs with P-NiO-0.5, NiO and Pt are depicted in Figure 7a. The ratio of the electrons generated in the external circuit at a specific wavelength to the incident photons under a short-circuit state is known as the IPCE of a dye-sensitized solar cell, and it is written as follows:

(4) $$\mathrm{IPCE}(\%)=\frac{\;1240\times J_{sc}}{\lambda \times \phi }$$

where λ is the wavelength, *J_sc_* is the short-circuit photocurrent density (mA cm^−2^) at the specific wavelength, and ϕ is the incident radiation flux (mW cm^−2^). The short-circuit current density value (*J_sc_*_-IPCE_) of a DSSC can also be calculated from its IPCE spectra, by integrating the area under the IPCE curve. The *J_sc_*_-IPCE_ values of the cells with P-NiO-0.5, NiO and Pt are shown in Table 3; these values of all the three DSSCs agree well with the corresponding *J_sc_* values.

### 3.8. Photovoltaic Performance of the DSSCs at Dim Lights

When the DSSC with P-NiO-0.5 CE was illuminated at various light intensities ranging from 20 to 100 mW cm^−2^, the η of the cell remained at about 99, 97, 90 and 87% of its initial value at 80, 60, 40,] and 20 mW cm^−2^ light conditions, respectively (Figure 7b and Table S2). It is notable that the η slowly decreases when the light intensity decreases dramatically. The performance of the DSSC with Pt CE remained at about 99, 96, 88 and 73% of its initial value at 80, 60, 40 and 20 mW cm^−2^ light conditions, respectively (Figure 7c and Table S3). This renders P-NiO-0.5 CE better than Pt for use as the electro-catalytic material in DSSCs, even for use in areas with less or oblique sunlight.

## 4. Conclusions

In this study, in order to replace the pricey typical Pt CE in DSSCs, a P-NiO electro-catalytic film in the shape of a flower was synthesized and incorporated into DSSCs. XPS revealed the chemical composition of P-NiO. From FE-SEM images, P-NiO-1 is viewed as an aggregation of many shattered fragments, and the P-NiO-0.75 is shown as broken nanosheets. P-NiO-0.5, P-NiO-0.25 and P-NiO-0.1, with a flower-like morphology of the nanosheets, exhibit higher surface areas to enhance the electro-catalytic activity toward I_3_^−^ reduction. The DSSC with the P-NiO-0.5 CE achieves the best η of 9.05%, while the cell with the Pt CE shows an η of 8.51%, indicating that the catalytic film of P-NiO is much superior to that of Pt. The DSSC with bare NiO exhibits only an η of 0.19%, owing to its poor intrinsic conductivity and very poor electro-catalytic ability. It is concluded that the doped P offers the catalytic ability of NiO in a multi-fold way. For rear illumination, the DSSC with P-NiO-0.5 CE shows the highest η of 5.4% among three comparable CEs. Through CV curves, the higher *J_pc_* and lower ΔE_p_ values suggest that the P-NiO-0.5 CE has superior electro-catalytic ability. The Tafel plot and EIS analysis of the P-NiO-0.5 CE agree well with the photovoltaic parameters of the pertinent DSSCs, since both have the smallest R_ct_ values. The cell of P-NiO-0.5 CE achieves a better performance than that of Pt CE regardless of weaker light conditions. The fact that the DSSC with P-NiO-0.5 CE offers a better performance than that with Pt CE suggests that the low-cost CE with P-NiO-0.5 is an attractive replacement for the DSSCs with Pt CE. Moreover, P-NiO–based electrodes can be feasibly be fabricated by a spray-coating process for practical application on a large scale.

## Figures and Tables

**Table 1:** Photovoltaic parameters of the DSSCs with CEs having various P-NiO films, measured at 100 mW cm^−2^ (AM1.5G) in front illumination. The standard deviation for the data is based on three cells.

CEs	*η* (%)	*V_oc_* (V)	*J_sc_* (mA cm^−2^)	*FF*
P-NiO-1	7.95 ± 0.02	0.77 ± 0.01	15.83 ± 0.04	0.65 ± 0.00
P-NiO-0.75	8.10 ± 0.03	0.77 ± 0.01	15.90 ± 0.01	0.66 ± 0.00
P-NiO-0.5	9.05 ± 0.04	0.79 ± 0.00	16.96 ± 0.01	0.68 ± 0.00
P-NiO-0.25	8.67 ± 0.08	0.79 ± 0.01	16.73 ± 0.04	0.65 ± 0.01
P-NiO-0.1	8.54 ± 0.03	0.79 ± 0.03	16.50 ± 0.07	0.66 ± 0.00

**Table 2:** Photovoltaic parameters of the DSSCs with the P-NiO-0.5, NiO and Pt CEs, measured at 100 mW cm^−2^ (AM1.5G) in front illumination. The standard deviation for the data is based on three cells.

CEs	*η* (%)	*V_oc_* (V)	*J_sc_* (mA cm^−2^)	*FF*
P-NiO-0.5	9.05 ± 0.04	0.79 ± 0.00	16.96 ± 0.01	0.68 ± 0.00
NiO	0.19 ± 0.00	0.76 ± 0.08	4.24 ± 0.04	0.06 ± 0.00
Pt	8.51 ± 0.00	0.79 ± 0.02	16.17 ± 0.03	0.67 ± 0.00

**Table 3:** Electrochemical parameters of the DSSCs with P-NiO-0.5, NiO and Pt CEs.

CEs	*J_pc_* (mA cm^−2^)	ΔE_p_ (V)	*J*_0_ (mA cm^−2^)	R_ct-Tafel_ (Ω cm^2^)	R_s_ (Ω cm^2^)	R_ct-EIS_ (Ω cm^2^)	*J_sc_*_-IPCE_ (mA cm^−2^)
P-NiO-0.5	1.20	0.38	3.37	3.81	23.54	3.87	13.84
NiO	N/A	N/A	4.27 × 10^−3^	3011	30.36	3258	3.33
Pt	0.91	0.47	2.71	4.74	19.71	4.51	13.25

## Data Availability

Not applicable.

## Supplementary Materials

The following supporting information can be downloaded at: https://www.mdpi.com/article/10.3390/nano12224036/s1. Table S1: Photovoltaic parameters of the DSSCs with P-NiO-0.5, NiO, and Pt CEs, measured at 100 mW cm^−2^ (AM1.5G) in rear illumination. The standard deviation for each data is based on three cells; Table S2: Photovoltaic parameters of the DSSC with P-NiO-0.5 CE, obtained at different light conditions. The standard deviation for each data is based on three cells; Table S3: Photovoltaic parameters of the DSSCs with Pt CE obtained at different light conditions. The standard deviation for each data is based on three cells; Figure S1: EDAX analysis of the P-NiO film; Figure S2: XPS spectra of the P-NiO in O 1s region; Figure S3: FE-SEM images of (a) P-NiO-1 and (b) P-NiO-0.75 before annealing process; Figure S4: FE-SEM images of cross section of (a) P-NiO-1, (b) P-NiO-0.75 (c) P-NiO-0.5, (d) P-NiO-0.25, and (e) P-NiO-0.1; Figure S5: Transmittance spectra of various films.
