# Supplementary material for: Nanoflower-like P-doped Nickel Oxide as a Catalytic Counter Electrode for Dye-Sensitized Solar Cells
Source: Nanomaterials (Basel). 2022 Nov 17;12(22):4036. doi: 10.3390/nano12224036 (PMC9692408; doi:10.3390/nano12224036)
Supplement: Supplementary file 1 [file nanomaterials-12-04036-s001.zip › nanomaterials-1974275-supplementary.pdf]

nanomaterials

Supplementary Information

# Nanoflower-Like P-Doped Nickel Oxide as a Catalytic Counter Electrode for Dye-Sensitized Solar Cells

**Yi-Lin Chen [1], Yi-June Huang [1], Min-Hsin Yeh [2,*], Miao-Syuan Fan [1], Cheng-Tai Lin [1], Ching-Cheng Chang [2], Vittal Ramamurthy [1] and Kuo-Chuan Ho [1,3,*]**

[1] Department of Chemical Engineering, National Taiwan University, Taipei 10617, Taiwan
[2] Department of Chemical Engineering, National Taiwan University of Science and Technology, Taipei 10607, Taiwan
[3] Institute of Polymer Science and Engineering, National Taiwan University, Taipei 10617, Taiwan
* Correspondence: mhyeh@mail.ntust.edu.tw (M.-H.Y.); kcho@ntu.edu.tw (K.-C.H.); Tel.: +886-2-2737-6643 (M.-H.Y.); +886-2-3366-3020 (K.-C.H.); Fax: +886-2-2737-6644 (M.-H.Y.); +886-2-2362-3040 (K.-C.H.)

**Table S1.** Photovoltaic parameters of the DSSCs with P-NiO-0.5, NiO, and Pt CEs, measured at 100 mW cm$^{-2}$ (AM1.5G) in rear illumination. The standard deviation for each data is based on three cells.

| CEs | $\eta$ (%) | $V_{oc}$ (V) | $J_{sc}$ (mA cm$^{-2}$) | *FF* |
|---|---|---|---|---|
| P-NiO-0.5 | 5.45 ± 0.03 | 0.76 ± 0.03 | 10.15 ± 0.07 | 0.71 ± 0.00 |
| NiO | 0.16 ± 0.00 | 0.73 ± 0.06 | 3.18 ± 0.02 | 0.07 ± 0.00 |
| Pt | 4.99 ± 0.02 | 0.76 ± 0.02 | 9.25 ± 0.01 | 0.71 ± 0.00 |

**Table S2.** Photovoltaic parameters of the DSSC with P-NiO-0.5 CE, obtained at different light conditions. The standard deviation for each data is based on three cells.

| Light Intensity (mW cm$^{-2}$) | $\eta$ (%) | $V_{oc}$ (V) | $J_{sc}$ (mA cm$^{-2}$) | *FF* |
|---|---|---|---|---|
| 100 (1 Sun) | 9.05 ± 0.04 | 0.79 ± 0.00 | 16.96 ± 0.01 | 0.68 ± 0.00 |
| 80 (0.8 Sun) | 8.97 ± 0.01 | 0.78 ± 0.01 | 13.13 ± 0.01 | 0.70 ± 0.00 |
| 60 (0.6 Sun) | 8.75 ± 0.02 | 0.76 ± 0.00 | 9.40 ± 0.01 | 0.73 ± 0.01 |
| 40 (0.4 Sun) | 8.21 ± 0.01 | 0.75 ± 0.00 | 5.87 ± 0.08 | 0.75 ± 0.00 |
| 20 (0.2 Sun) | 7.87 ± 0.00 | 0.73 ± 0.02 | 2.86 ± 0.01 | 0.75 ± 0.00 |

**Table S3.** Photovoltaic parameters of the DSSCs with Pt CE obtained at different light conditions. The standard deviation for each data is based on three cells.

| **Light intensity (mW cm$^{-2}$)** | $\eta$ **(%)** | $V_{oc}$ **(V)** | $J_{sc}$ **(mA cm$^{-2}$)** | ***FF*** |
|---|---|---|---|---|
| 100 (1 Sun) | 8.51 ± 0.00 | 0.79 ± 0.00 | 16.17 ± 0.01 | 0.67 ± 0.00 |
| 80 (0.8 Sun) | 8.47 ± 0.01 | 0.77 ± 0.01 | 12.41 ± 0.01 | 0.70 ± 0.00 |
| 60 (0.6 Sun) | 8.16 ± 0.00 | 0.76 ± 0.00 | 9.01 ± 0.03 | 0.71 ± 0.00 |
| 40 (0.4 Sun) | 7.51 ± 0.01 | 0.75 ± 0.00 | 5.61 ± 0.04 | 0.72 ± 0.01 |
| 20 (0.2 Sun) | 6.17 ± 0.05 | 0.71 ± 0.00 | 2.66 ± 0.03 | 0.65 ± 0.00 |

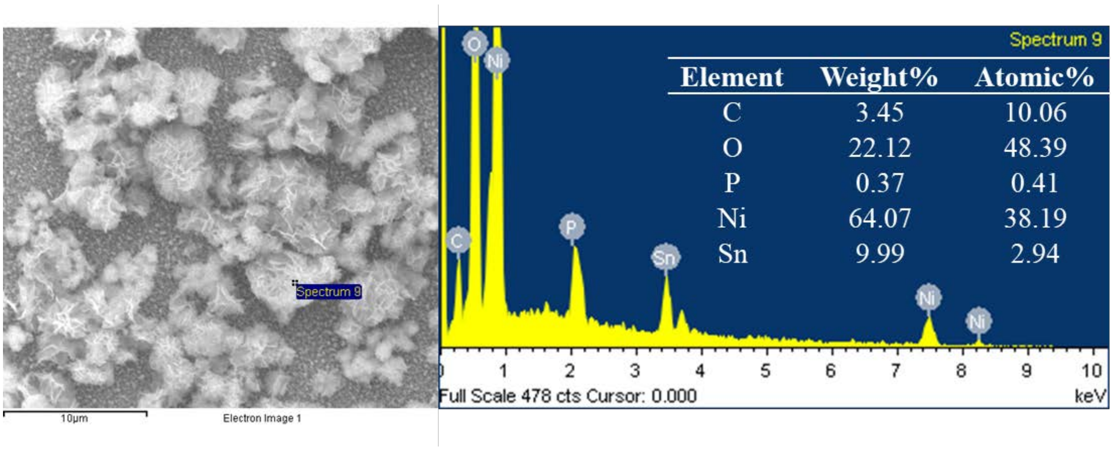


**Figure S1.** EDAX analysis of the P-NiO film.

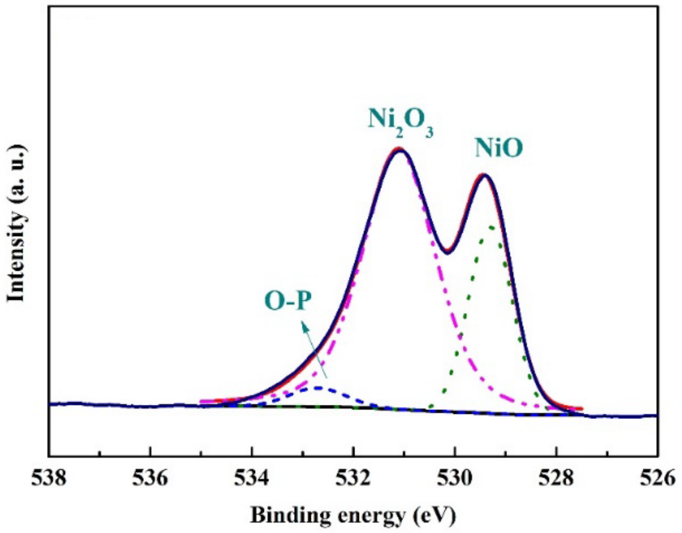


**Figure S2.** XPS spectra of the P-NiO in O 1s region.

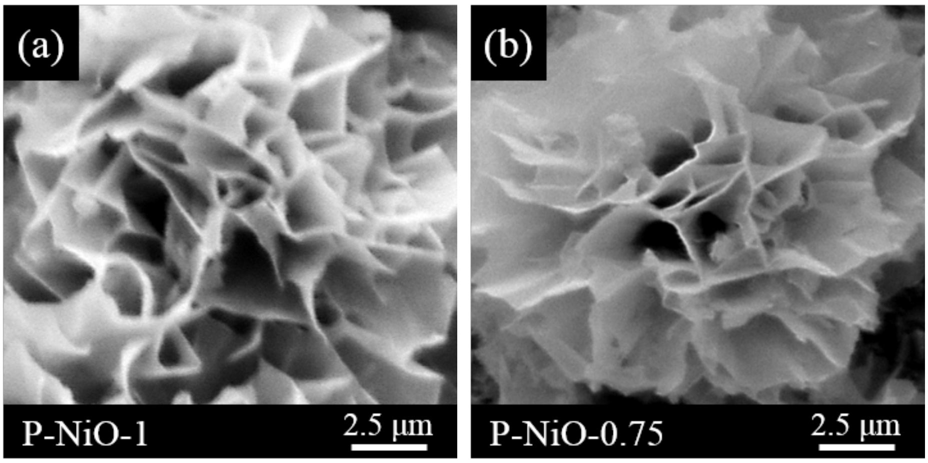


**Figure S3.** FE-SEM images of (**a**) P-NiO-1 and (**b**) P-NiO-0.75 before annealing process.

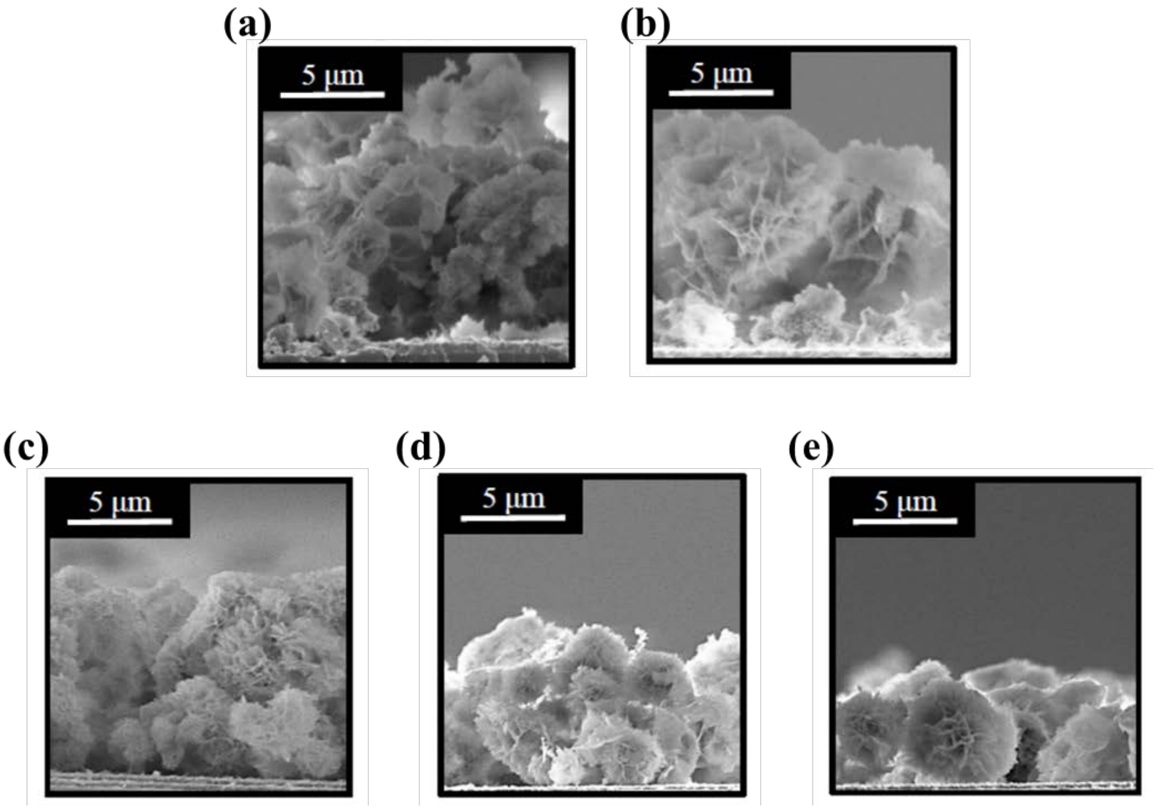


**Figure S4.** FE-SEM images of cross section of (**a**) P-NiO-1, (**b**) P-NiO-0.75 (**c**) P-NiO-0.5, (**d**) P-NiO-0.25, and (e) P-NiO-0.1.

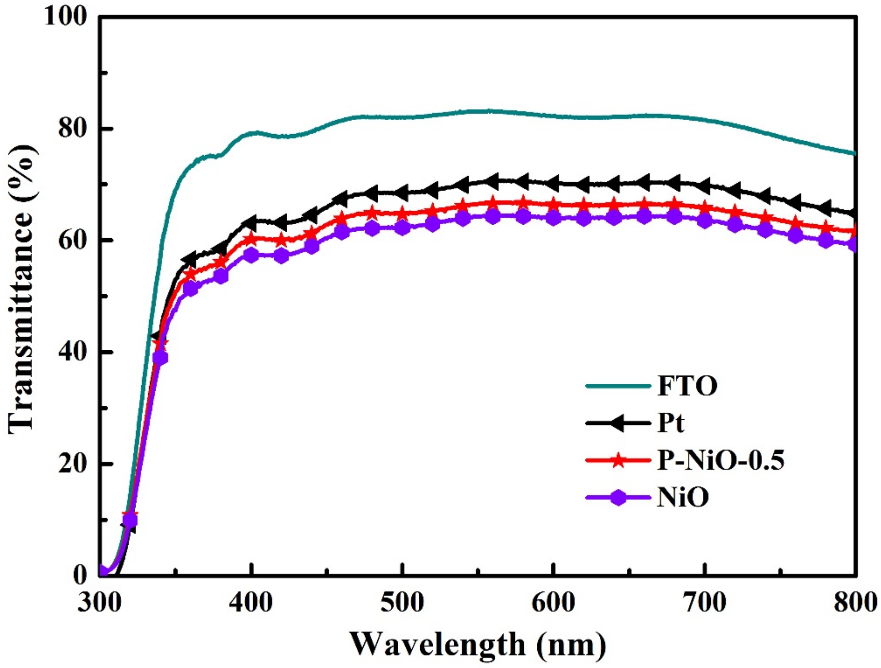


**Figure S5.** Transmittance spectra of various films.
